# Existence of intratumoral tertiary lymphoid structures is associated with immune cells infiltration and predicts better prognosis in early-stage hepatocellular carcinoma

**DOI:** 10.18632/aging.102821

**Published:** 2020-02-22

**Authors:** Hui Li, Jinju Wang, Hailing Liu, Tian Lan, Lin Xu, Genshu Wang, Kefei Yuan, Hong Wu

**Affiliations:** 1 Department of Liver Surgery, Liver Transplantation Division, West China Hospital, Sichuan University, Chengdu 610041, China; 2 Laboratory of Liver Surgery, West China Hospital, Sichuan University, Chengdu 610041, China; 3 Department of Hepatic Surgery and Liver Transplantation Center, The Third Affiliated Hospital of Sun Yat-Sen University, Guangzhou 510006, China

**Keywords:** hepatocellular carcinoma, intratumoral tertiary lymphoid structures, early relapse, immune response, immunotherapy

## Abstract

Tumor-associated tertiary lymphoid structures (TLS) play a critical role in the progression of various tumors. However, the dynamics of lymphocyte recruitment during hepatocellular carcinoma (HCC) clinical progression have not been fully elucidated. In the present study, tissue microarrays and hematoxylin-eosin staining were used to evaluate the existence and degree of TLS in HCC patients. Nine immune biomarkers in intratumoral tissues were examined by immunohistochemical staining. A total of 462 patients were recruited for the study. Kaplan–Meier analysis showed that TLS was inversely correlated with the risk of early tumor recurrence (P=0.014), whereas no association was found between TLS and overall survival. Cox regression analysis identified TLS as an independent prognostic factor for early HCC recurrence (P=0.005). In addition, TLS was associated with increased intratumoral CD3+, CD8+, CD20+, and decreased infiltration of Foxp3+ and CD68+ cells. A lower density of PD1+, TIM3+, and LAG3+ were found in TLS+ cases. Sub-analysis revealed the prognostic value of TLS on early-stage HCC (BCLC 0-A, TNM stage I-II) and HCC with solitary nodule. The validation cohort verified the prognostic value of TLS in early-stage HCC patients. These results suggest that TLS-targeted immune-modulating therapies may be a potential strategy for effective immune-mediated tumor suppression.

## INTRODUCTION

Hepatocellular carcinoma (HCC) is the sixth most common cancer and the fourth leading cause of malignancy-related mortality worldwide [1]. Among the currently available therapeutic strategies, surgical interventions such as hepatectomy and liver transplantation remain the mainstay of HCC treatment [1, 2]. However, HCC prognosis is still unsatisfactory with a 5-year survival rate of 60%-80% for well-selected candidates following curative resection [3]. Besides, HCC is one of the most frequently chemo-resistant tumor types [1]. For advanced HCC, sorafenib is still the standard therapeutic drug [4, 5].

Recently, immunotherapy has attracted special attention, which can be attributed to the success of checkpoint inhibitors in various solid and hematological malignancies [6, 7]. Studies have liked tumor microenvironment (TME) with tumorigenesis and progression. Tumor-infiltrating lymphocytes occur in various tumors and therefore act as prognostic markers for higher responses to chemotherapy and better survival [8–10]. Studies on tumor-associated lymphocytes have suggested the formation of tertiary lymphoid structures (TLS) as a potential antitumor immune response [11–13]. Also, tertiary lymphoid structure describes ectopic lymphoid formations commonly observed in allograft rejection, autoimmune diseases and tumoral tissues [11, 14]. It exhibits all the characteristics of formation in normal lymph nodes [11]. Previous studies have associated the occurrence of TLS with decreased risk of recurrence and better overall survival (OS) in various solid tumors [15, 16].

However, their prognostic value in HCC remains controversial. Finkin et al. reported a positive correlation between the presence of non-tumoral TLS and poor prognosis of HCC patients treated with hepatectomy [17]. Contrarily, Calderaro et al. found that intratumoral TLS were associated with a reduced risk of early recurrence of HCC after surgical resection [18].

Given that many kinds of immune cells are involved in TME, it is important to identify specific immune cells that are recruited into tumor tissues during immune response and immunosuppression. The present study aimed to verify the prognostic value of intratumoral TLS in post hepatectomy HCC patients. To achieve this, we conducted a morphological analysis and examined the expression levels of immune markers to reveal the correlation between the existence of intratumoral TLS and immune infiltration.

## RESULTS

### Clinicopathological characteristics of patients

**Figure 1:**
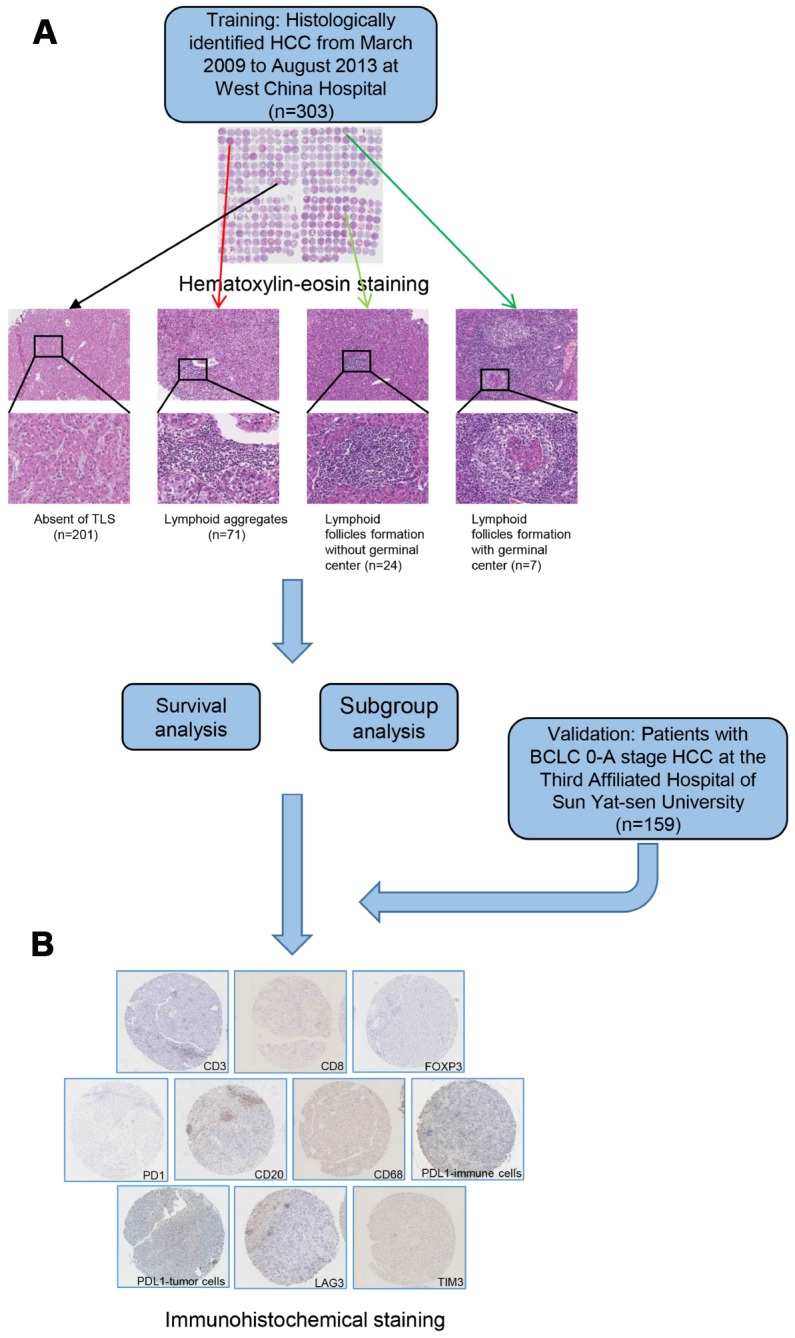
**A flow diagram showing the characterization of intratumoral TLS.** Patients were classified into 4 groups according to hematoxylin-eosin staining (**A**) absence of TLS; presence of lymphoid aggregates; intratumoral lymphoid follicles without germinal center; and intratumoral lymphoid follicles with germinal center. Samples from patients in training and validation cohort were examined by immunohistochemical staining (**B**). TLS, tertiary lymphoid structures.

A total of 303 surgically treated HCC patients were enrolled in the training cohort and 159 patients in the validation cohort. The demographic and clinicopathological features are summarized in Table 1. In the training cohort, 102 (33.7%) patients were TLS positive (TLS+), whereas 201 were TLS negative (TLS-). Among the 102 TLS+ cases, lymphoid follicles were observed in 19 cases (18.6%) and lymphoid aggregates were presented in 83 (81.4%) cases (Figure 1). The association between TLS existence and clinicopathological characteristics was detected (Supplementary Table 2). At median follow-up time of 61.3 months (range, 1.5-119.4 months), 207 (68.3%) patients experienced tumor relapse. A recurrence rate of 29.7% (90 patients) was recorded within 2 years after hepatic resection. The TLS positive (TLS+) patients were associated with a decreased risk of early tumor recurrence (19.6% versus 34.8%, P=0009). However, the 5-year survival rate was comparable between the two groups. In the training cohort, 79.9% (242/303) of the patients underwent hepatic resection for BCLC stage 0-A HCC. The correlation between their clinicopathological features and the occurrence of TLS is summarized in Supplementary Table 3. Patients with early-stage HCC were enrolled in the validation cohort (n=159). No significant heterogeneity was observed among patients with BCLC stage 0-A HCC from the training cohort and patients in validation cohort (Supplementary Table 4). Consistently, apart from early tumor recurrence, TLS was not linked to any other clinical, biological, or pathological features (Supplementary Table 5). Hematoxylin-eosin staining was performed to examine the occurrence of TLS in an extra cohort of patients who underwent hepatectomy for hepatic hemangioma (n=50). It is noteworthy that only lymphoid aggregates were observed in four of them, whereas no lymphoid follicles were found.

**Table 1:** Clinicopathological characteristics of patients in training and validation cohort.

**Variables**	**Training cohort (*n*=303)**	**Validation cohort (n=159)**	***P* Value**
Age (year)	51.1±12.5	51.2±12.6	0.996
Gender (M/F)	251/52	132/27	0.961
HBsAg (+/-)	265/38	140/19	0.854
HBV DNA (0/10^3^-10^5^/>10^5^)	97/105/101	50/57/52	0.931
HCV infection (+/-)	6/297	3/156	0.945
Cirrhosis (+/-)	190/113	98/61	0.821
Portal hypertension (+/-)	42/261	22/137	0.994
Ascites (+/-)	36/267	20/139	0.827
AFP (≥400/<400) (ng/dL)	124/179	64/95	0.889
Tumor size (≥5cm/<5cm)	166/137	84/75	0.689
Tumor number (multiple/single)	52/251	0/159	N/A
Differentiation (Poor /Well-Moderate)	127/176	68/91	0.921
Macrovascular invasion	13 (4.3%)	0 (0%)	N/A
Microvascular invasion	105 (34.7%)	50 (31.4%)	0.488
BCLC stages (B-C/0-A)	61/242	0/159	N/A
TNM stages (III-IV/I-II)	76/227	22/137	0.005
Early recurrence	90 (29.7%)	45 (28.3%)	0.178
5-year survival	162 (53.5%)	80 (50.3%)	0.519

F: female; M: male; HBV: hepatitis B virus; HCV: hepatitis C virus; AFP: alpha-fetoprotein; BCLC: Barcelona Clinic of Liver Cancer; TNM: tumor-nodes-metastasis; TLS: tertiary lymphoid structures; N/A: not available.

### Association between TLS and tumor recurrence

**Figure 2:**
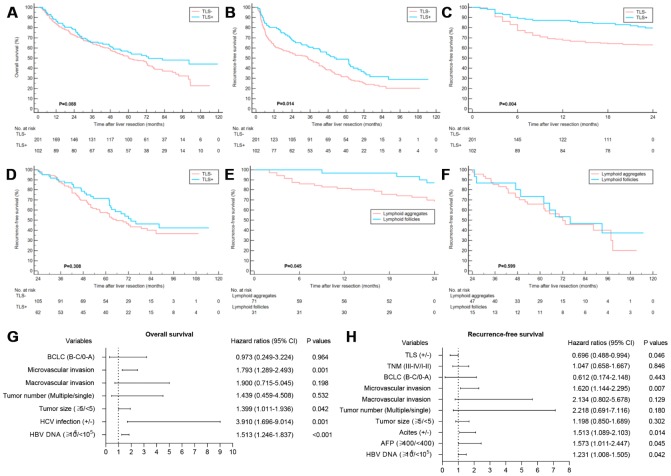
**Clinical relevance of TLS in training cohort.** (**A**) Kaplan–Meier curves showing no significant correlation between the occurrence of TLS and OS (*P*=0.088); (**B**) Kaplan–Meier curves showing patients with TLS+ had better RFS (*P*=0.014); (**C** and **D**) Kaplan–Meier curves showing patients with TLS+ had better early RFS (*P*=0.004) and comparable late RFS (*P*=0.308); (**E** and **F**) Kaplan–Meier curves showing patients with lymphoid follicles had better early RFS (*P*=0.045) and comparable late RFS (*P*=0.599) relative to those with lymphoid aggregates; (**G**) Multivariate analyses for OS; (**H**) Multivariate analyses showed that TLS was an independent predictor of RFS. TLS, tertiary lymphoid structures; OS, overall survival; RFS, recurrence-free survival.

**Figure 3:**
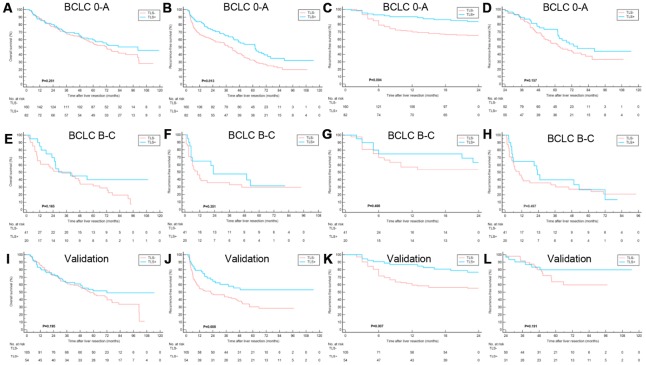
**Clinical relevance of TLS in training cohort and validation cohort subgroups.** (**A**–**D**) Kaplan–Meier curves showing that in patients with BCLC 0-A stage HCC, TLS+ was correlated to similar OS, better RFS, better early RFS and similar late RFS compared to those with TLS-; (**E**–**H**) Kaplan–Meier curves showing no significant correlation between TLS and OS, RFS, early RFS and late RFS for patients with BCLC B-C stage HCC; (**I**–**L**) Kaplan–Meier curves showing patients with TLS+ had similar OS, better RFS, better early RFS and similar late RFS compared to those with TLS- in the validation cohort. TLS, tertiary lymphoid structures; OS, overall survival; RFS, recurrence-free survival; BCLC, Barcelona Clinic of Liver Cancer.

The TLS- patients were associated with the worse RFS, whereas no significant correlation was found between the existence of TLS and OS (Figure 2A, 2B). Notably, TLS was statistically correlated with early tumor relapse other than late tumor recurrence (Figure 2C, 2D). Also, patients with lymphoid follicles had a lower risk of early recurrence than those with only lymphoid aggregates (P=0.045) (Figure 2E, 2F), suggesting that the degree of TLS maturation had a prognostic impact. The potential features which were correlated with OS and RFS in univariate analysis are summarized in Supplementary Table 6. Multivariate analysis identified elevated HBV-DNA, HCV infection, larger tumor size and microvascular invasion as independent prognosticators for OS. The intratumoral TLS was an independent favorable factor for early relapse (Figure 2G, 2H). Univariate and multivariate Cox regression analyses were performed for early and late RFS to further evaluate the prognostic effect of intratumoral TLS. The results showed TLS was an independent predictor of early other than late tumor relapse (Table 2). The results of the Cox regression model analysis of the early-stage HCC patients in the training and validation cohorts were consistent with the results of the entire cohort (Figure 3, Table 3 and Supplementary Tables 7, 8).

**Table 2:** Analysis for early and late recurrence-free survival using the univariate and multivariate Cox proportional hazards regression model in the training cohort.

**Variables**	**Early recurrence-free survival**				**Late recurrence-free survival**			
	**Univariate analysis**		**Multivariate analysis**		**Univariate analysis**		**Multivariate analysis**	
	**HR (95%CI)**	**P value**	**HR (95%CI)**	**P value**	**HR (95%CI)**	**P value**	**HR (95%CI)**	**P value**
Age	0.985 (0.969-1.001)	0.072			1.010 (0.994-1.027)	0.229		
Gender (F/M)	0.503 (0.253-1.002)	0.051			0.766 (0.441-1.331)	0.345		
HBsAg (+/-)	1.432 (0.719-2.852)	0.307			1.219 (0.665-2.234)	0.522		
HBV DNA (>10^5^/10^3^-10^5^/0)	1.323 (1.023-1.712)	0.033	1.222 (0.939-1.591)	0.136	1.109 (0.860-1.430)	0.425		
HCV infection (+/-)	1.609 (0.296-6.537)	0.506			0.895 (0.125-6.424)	0.912		
Cirrhosis (+/-)	0.816 (0.537-1.240)	0.341			1.413 (0.916-2.181)	0.118		
Portal hypertension (+/-)	0.971 (0.529-1.783)	0.924			1.519 (0.909-2.540)	0.111		
Ascites (+/-)	2.004 (1.160-3.461)	0.013	1.865 (1.086-3.202)	0.024	1.559 (0.868-2.800)	0.137		
AFP (≥400/<400)	1.969 (1.301-2.982)	0.001	1.735 (1.127-2.669)	0.012	1.438 (0.955-2.166)	0.082		
Tumor size (≥5/<5)	2.259 (1.438-3.547)	<0.001	1.688 (1.048-2.717)	0.031	1.255 (0.837-1.883)	0.271		
Tumor number (multiple/single)	1.594 (0.960-2.646)	0.071			1.757 (1.051-2.937)	0.032	1.167 (0.270-5.045)	0.836
Differentiation (Poor /Well-Moderate)	0.926 (0.607-1.412)	0.721			1.084 (0.718-1.636)	0.703		
Macrovascular invasion	2.627 (1.212-5.692)	0.014	1.034 (0.412-2.597)	0.943	N/A	N/A		
Microvascular invasion	2.240 (1.480-3.389)	<0.001	1.648 (1.043-2.604)	0.032	1.692 (1.104-2.592)	0.016	1.571 (1.014-2.435)	0.043
BCLC (B-C/0-A)	1.673 (1.041-2.688)	0.033	1.221 (0.696-2.144)	0.486	1.762 (1.075-2.888)	0.025	1.377 (0.334-5.676)	0.658
TNM (III-IV/I-II)	1.271 (0.801-2.015)	0.309			1.465 (0.927-2.316)	0.102		
TLS (+/-)	0.485 (0.295-0.797)	0.004	0.501 (0.302-0.830)	0.007	0.804 (0.521-1.241)	0.326		

F: female; M: male; HBV: hepatitis B virus; HCV: hepatitis V virus; AFP: alpha-fetoprotein; BCLC: Barcelona Clinic of Liver Cancer; TNM: tumor-nodes-metastasis; TLS: tertiary lymphoid structures; HR, hazard ratio; CI, confidence interval; N/A: not available (cases were too little).

**Table 3:** Analysis for overall survival and recurrence-free survival using the univariate and multivariate Cox proportional hazards regression model for BCLC stage 0-A HCC in the validation cohort.

**Variables**	**Overall survival**				**Recurrence-free survival**			
**Univariate analysis**		**Multivariate analysis**		**Univariate analysis**		**Multivariate analysis**	
**HR (95%CI)**	**P value**	**HR (95%CI)**	**P value**	**HR (95%CI)**	**P value**	**HR (95%CI)**	**P value**
Age	0.999(0.983-1.015)	0.902			1.006(0.990-1.022)	0.496		
Gender (F/M)	0.831(0.477-1.447)	0.513			0.633(0.352-1.138)	0.126		
HBsAg (+/-)	1.959(0.905-4.239)	0.088			1.148(0.612-2.153)	0.668		
HBV DNA (>10^5^/10^3^-10^5^/0)	1.586(1.212-2.075)	0.001	1.521(1.156-2.001)	0.003	1.206(0.932-1.560)	0.154		
Cirrhosis (+/-)	1.073(0.704-1.638)	0.742			0.899(0.594-1.360)	0.614		
Portal hypertension (+/-)	0.705(0.365-1.362)	0.298			1.197(0.689-2.080)	0.522		
Ascites (+/-)	1.497(0.846-2.648)	0.166			2.504(1.455-4.310)	0.001	2.550(1.452-4.480)	0.001
AFP (≥400/<400)	1.156(0.762-1.753)	0.496			1.501(0.998-2.256)	0.051		
Tumor size (≥5/<5)	2.070(1.350-3.175)	0.001	1.900(1.220-2.959)	0.005	1.808(1.193-2.740)	0.005	1.615(1.052-2.480)	0.028
Differentiation (Poor /Well-Moderate)	1.509(1.002-2.274)	0.049	1.426(0.941-2.161)	0.094	1.421(0.947-2.132)	0.090		
Microvascular invasion	1.742(1.139-2.665)	0.011	1.372(0.882-2.134)	0.161	2.048(1.353-3.101)	0.001	1.690(1.099-2.599)	0.017
TLS (+/-)	0.742(0.472-1.167)	0.196			0.534(0.334-0.855)	0.008	0.524(0.326-0.482)	0.008

F: female; M: male; HBV: hepatitis B virus; HCV: hepatitis V virus; AFP: alpha-fetoprotein; BCLC: Barcelona Clinic of Liver Cancer; TLS: tertiary lymphoid structures; HR, hazard ratio; CI, confidence interval.

**Figure 4:**
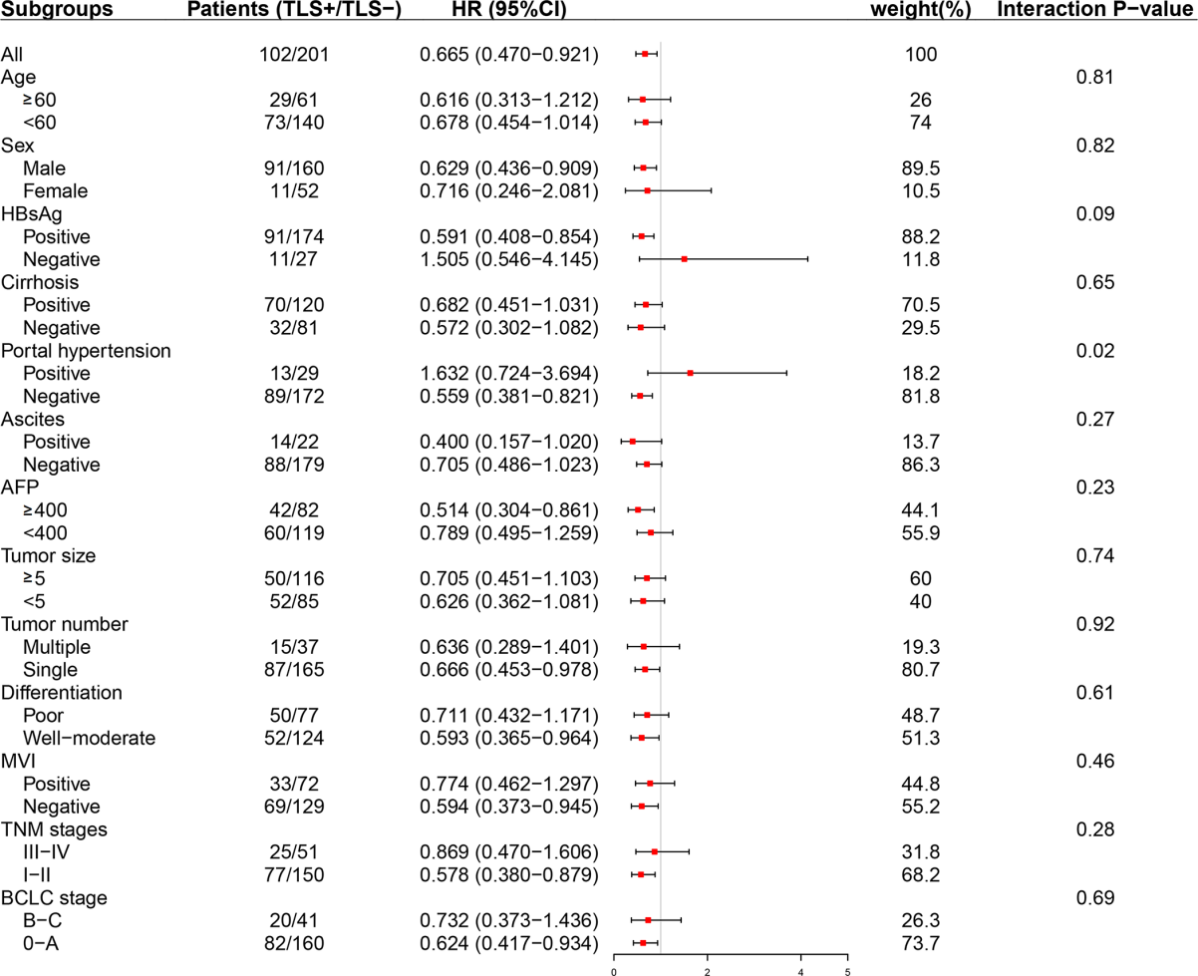
**Subgroup analysis based on clinicopathologic features (early RFS).** TLS was significantly correlated with early tumor relapse in patients with BCLC 0-A stage HCC other than those with advanced tumors. TLS, tertiary lymphoid structures; AFP, alpha-fetoprotein; BCLC, Barcelona Clinic of Liver Cancer; TNM, tumor-nodes-metastasis.

Subgroup analyses were conducted to evaluate the prognostic value of intratumoral TLS in patients stratified by potential sources of heterogeneity. Tumor-associated tertiary lymphoid structures (TLS) had a prognostic effect on early-stage (BCLC 0-A, TNM stage I-II) HCC as well as HCC with solitary nodule (Figure 4 and Supplementary Figures 1–3).

Furthermore, the area under the receiver operating characteristic (AUC) of TLS in predicting RFS and early tumor recurrence of patients in the training cohort were 0.64 and 0.715, respectively. For patients with BCLC 0-A stage HCC, the AUCs were 0.682 for RFS and 0.798 for early RFS, suggesting that TLS was a precise prognostic marker for early recurrence of early-stage HCC in patients (Supplementary Figure 4).

### TLS is associated with immune infiltration

**Figure 5:**
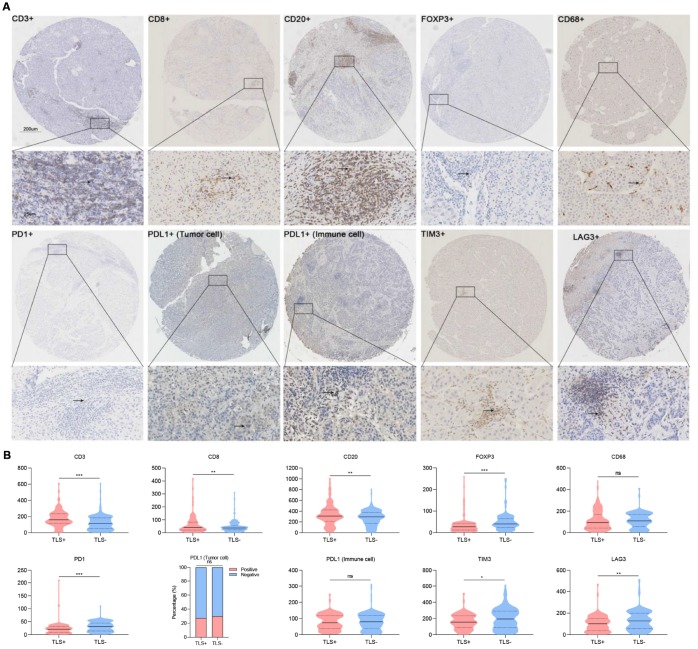
**Association between TLS and immune infiltration.** (**A**) Representative images showing immunohistochemical staining of the nine immune markers in immune cells (CD3, CD8, CD20, FOXP3, CD68, PD1, PDL1, TIM3, LAG3) and tumor cells (PDL1); (**B**) Statistical analyses showing TLS was associated with increased intratumoral CD3+, CD8+, CD20+ and decreased Foxp3+, CD68+ cells infiltration as well as lower density of PD1+, TIM3+ and LAG3+. TLS, tertiary lymphoid structures; *: P<0.05, **: P<0.01, ***: P<0.001, ns: no significance. Solid lines represent mean values, dotted lines represent quartiles.

After evaluating the levels of stained immune infiltrations, we found an increased tumor infiltration with CD3+ and CD8+ T cells in TLS+ cases. Also, more tumor-infiltrating CD20+ cells (representing B lymphocytes except for plasma cells) were observed in TLS+ HCC. Contrarily, the infiltration of Foxp3+ regulatory T cells (Foxp3+ Tregs) and macrophages (CD68+) was negatively correlated with TLS, and therefore their levels were significantly low in the TLS+ group (Figure 5).

Because multiple immunosuppressive mechanisms were involved in HCC, we detected expression levels of several immune checkpoint molecules in TME. A decreased number of PD1+ cells, TIM3+ cells, and LAG3+ cells were observed in TLS+ cases. However, no correlation was found between PD-L1+ tumor cells and PD-L1 immune cells in TLS+ cases (Figure 5).

## DISCUSSION

The aim of this study was to confirm the prognostic value of the intratumoral TLS in predicting the relapse of HCC in patients and to investigate its role in the prognosis of HCC patients after curative resection. Overall, the results of the present study showed that the existence and degree of TLS maturation were associated with decreased risk of early HCC recurrence, but this was not linked to OS and late tumor relapse. It is noteworthy that the prognostic value of TLS was limited to BCLC stage 0-A HCC treated with surgical resection. For advanced HCC (BCLC stage B-C), no association was observed between intratumoral TLS and OS or RFS).

Tumor-associated TLS comprise lymphocytes assembled at organs or tissues whose primary function is neither the initiation of an adaptive immune response nor the generation of immune structures [19]. They exhibit similar morphological, cellular, and molecular properties to secondary lymphoid organs, particularly lymphoid nodes [20]. Initially, TLS were only known to be present in non-neoplastic chronic inflammation, such as allograft rejection, autoimmune diseases and infections [21, 22]. Several studies have proposed that malignancies could preclude the formation of TLS because of the highly immunosuppressive tumor microenvironment [14, 23, 24]. However, the presence of tumor-associated TLS and various degrees of the TLS maturation has been observed in a variety of primary and metastatic malignancies [15, 25, 26]. Most of the studies on the functions of intratumoral TLS have suggested favorable clinical prognostic value in various solid tumors. Finkin et al. reported that the existence of TLS in non-tumoral liver parenchyma was indicative of an elevated risk of late recurrence as well as poor OS of HCC patients after surgical resection [17]. Contrarily, Calderaro et al. demonstrated that non-neoplastic liver associated TLS were not linked to early or late relapse of HCC but intratumoral TLS were associated with decreased risk of early tumor recurrence [18]. The results of this study verified the prognostic value of intratumoral TLS in predicting early relapse for BCLC stage 0-A HCC. No association between intratumoral TLS and OS or late tumor recurrence was observed. Also, intratumoral TLS formation had no prognostic value in BCLC stage B-C HCC.

The mechanism underlying the association between TLS and a favorable HCC prognosis has not been entirely illuminated. One theory holds that tumor-associated TLS may cause the tumor-infiltrating lymphocytes to foster antitumor immune responses. Patients with high CD8+ T cell infiltration in combination with a high density of tumor-associated TLS showed significantly better prognosis than those without TLS in non-small-cell lung cancer [27]. Kroeger et al. reported that TLS facilitated coordinated antitumor responses of tumor-associated plasma cells with tumor-infiltrating CD8+ T cells [28]. Moreover, sophisticated TLS with segregated B and T cell zones with germinal centers indicates favorable clinical outcomes [29]. Consistently, we observed a higher density of CD3+ and CD8+ lymphocytes in TLS+ cases. As the key component of humoral immunity, the prognostic effect of B cells in human solid tumors remains controversial. Faggioli et al. reported that a high density of infiltrating B cells was associated with tumor aggressiveness and poor survival [30]. On the contrary, Zhang et al. revealed a favorable role of tumor-infiltrating CD20+ B cells in HCC. In this study, TLS formation was correlated with increased intratumoral CD20+ B cells and superior survival in HCC [31]. Furthermore, evidence has associated tumor-infiltrating macrophages with tumor progression and poor survival [31]. The findings of the present study demonstrated a negative correlation between intratumoral CD68+ macrophages and the existence of intratumoral TLS. Germinal centers within lymphoid nodes are indicative of immune response activation, and therefore better survival outcomes, which further reveals the capacity of TLS in facilitating humoral immune responses [32]. However, in the present study, a separate analysis of lymphoid follicles with mature germinal center was unable to perform considering limited number was observed in the tissue arrays.

Apart from the immune response, immunosuppressive components within ectopic lymphoid structures confer the deleterious outcomes for tumor progression. Shields et al. reported that the recruitment of myeloid-derived suppressor cells and Foxp3+ Treg cells to B16 melanomas-associated TLS induced the secretion of CCL21, thereby facilitating the host immune tolerance and tumor progression [33]. Moreover, Tregs located in tumor mass are not significantly associated with tumor evolution, whereas Tregs within intratumoral TLS exhibits suppression of immune activation [34]. In this study, TLS was correlated with decreased density of intratumoral Foxp3+ Treg cells in HCC. Also, a decreased density of PD1+, TIM3+, and LAG3+ cells were observed in TLS+ cases. Several lines of evidence have suggested that these immune checkpoints could be a potential immune inhibitory mechanism by which cancer cells evade anti-tumor immunity [35]. However, in the present study, no significant association was found between the existence of TLS and the expression of PD-L1. Collectively, our results indicated that TLS served as active structures concerning immune responses, either as an effective antitumor immune activator or tumor microenvironment fostered immunosuppressor.

The location of TLS could be intratumoral and extratumoral, which is relative to tumor origin or disease stages [14]. Extratumoral TLS have been reported to indicate worse OS and increased risk of late tumor recurrence for HCC [17]. Several studies have associated the prognostic value of TLS with early rather than advanced stage tumors [36, 37]. Indeed, in parallel to the tumor progression, immunosuppressive tumor microenvironment tends to be established as well as reduction of tumor immunogenicity [14]. In the present work, the absence of intratumoral TLS was indicative of early tumor relapse for BCLC stage 0-A HCC, whereas no association between intratumoral TLS and prognosis of BCLC stage B-C HCC was observed.

Therefore, to induce an effective antitumor immune response, key signaling molecules targeting TLS should be included in therapies. Our work verified the prognostic value of intratumoral TLS in early-stage HCC. However, further studies are needed to investigate the underlying mechanism as well as clinical trials based on immune response components. Additionally, limitations of this study warrant consideration when interpreting our findings. Tissue arrays provide consistent conditions for immunohistochemistry as well as an increased risk of loss of the tumor tissues which were associated with TLS presence. Our analyses did not involve extratumoral TLS. Also, in the multivariate analysis for late tumor recurrence, the etiology of HCV infection was not included because of limited patients. Thus, further studies with large sample sizes are needed to identify the precise role of intratumoral or extratumoral TLS within pathological foci. This can be crucial in the development of effective therapeutic targets.

In summary, the present study suggested that the existence of intratumoral TLS was associated with decreased risk of early tumor recurrence for HCC patients after hepatic resection. There was no significant association between intratumoral TLS and OS as well as late tumor relapse. Notably, the prognostic value of intratumoral TLS was identified in the BCLC stage 0-A HCC but not in advanced stages of the tumor. The TLS formation is a complex process involving antitumor immune activation and immunosuppression. The development of TLS-targeted immune-modulating therapies may be a potential strategy for effective immune-mediated tumor suppression.

## MATERIALS AND METHODS

### Study population

Patients who underwent hepatectomy for newly diagnosed HCC at West China Hospital from March 2009 to August 2013 were retrospectively enrolled as the training cohort. The validation cohort included patients who underwent hepatic resection for BCLC (Barcelona Clinic of Liver Cancer) stage 0-A HCC at the Third Affiliated Hospital of Sun Yat-sen University. The following clinicopathological features were reviewed and recorded from the hospital’s handwritten or electronic medical records: basic information (sex, age), hepatitis B virus (HBV) infection, hepatitis C virus (HCV) infection, other etiologies, cirrhosis (detected using ultrasound or imageological examination), ascites, BCLC stage, tumor-nodes-metastasis (TNM) stage and preoperative serum alpha-fetoprotein (AFP) level. For patients infected HBV, quantification for HBV-DNA was routinely performed. Also recorded were the following tumor characteristics: number of tumors, diameter of largest tumor, macrovascular invasion, pathologically microvascular invasion and tumor differentiation. This study was approved by the ethics committee of the West China Hospital and the Third Affiliated Hospital of Sun Yat-sen University. And was conducted as per the guidelines of the 1975 Declaration of Helsinki [38]. Patients were followed up according to National Comprehensive Cancer Network (NCCN) guidelines [39]. Besides, telephone follow-up survey was used to contact patients who could not go back to the hospital for reexamination.

### Tissue microarray, immunohistochemistry and evaluation of staining cells

Briefly, HCC tissue specimens were buffered, fixed using formalin, then embedded in paraffin for microarray analysis. Tissue microarrays were prepared as previously described [40, 41]. An experienced pathologist specialized in liver diseases reviewed the tissue arrays after hematoxylin and eosin staining. The existence of tumor-associated tertiary lymphoid structures (TLS) was assessed based on a scale described previously [17, 18, 42]. The TLS were divided into three main grades: lymphoid aggregates, lymphoid follicles formation without germinal center and lymphoid follicles formation with germinal center [18]. As previously described, tumors with at least one observable TLS were defined as intratumoral TLS+, whereas tumors without any observable TLS were defined as TLS- [18].

Nine immune markers (CD3, CD8, CD20, Foxp3, CD68, PD1, PD-L1, TIM3, and LAG3) were selected for staining in this study because of their involvement in tumor prognosis as previous studies and our previous work described [43]. The detailed information of antibodies and staining conditions for immunohistochemical staining (IHC) are summarized in Supplementary Table 1.

The levels of stained immune infiltrations were evaluated using three most representative areas (photographed at × 200 magnification). The numbers of positive cells were counted and converted to cell density (cells/mm^2^). Mean values were used for statistical analysis. Specially, PD-L1-positive tumor were those with more than 1% staining on their membranes.

### Statistical analysis

The SPSS (version 23.0) and MedCalc (version 15.2.2) software were used to perform the statistical analyses. Categorical variables were analyzed by Chi-square test and Fisher’s exact test, whereas continuous variables were evaluated by student’s t-test and Kruskal-Wallis test. The Monte Carlo method was used to assess multiple hypothesis test. The survival curves were plotted using Kaplan-Meier method and tested by log-rank test. Subsequently, Cox proportional hazards regression model (enter method) was employed to identify potential independent prognostic factors for overall survival (OS) and recurrence free survival (RFS). Potential confounders that were correlated to survival outcomes and had P values less than 0.05 in univariate regression analyses were selected for multivariate regression models. The receiver operating characteristic (ROC) curve was used to evaluate the accuracy of TLS in predicting tumor recurrence. A two-tailed P <0.05 was considered statistically significant.

## Supplementary Material

Supplementary Figures

Supplementary Tables
