# Supplementary material for: Existence of intratumoral tertiary lymphoid structures is associated with immune cells infiltration and predicts better prognosis in early-stage hepatocellular carcinoma
Source: Aging (Albany NY). 2020 Feb 22;12(4):3451–72. doi: 10.18632/aging.102821 (PMC7066901; doi:10.18632/aging.102821)
Supplement: Supplementary Figures [file aging-12-102821-s002..pdf]

SUPPLEMENTARY FIGURES

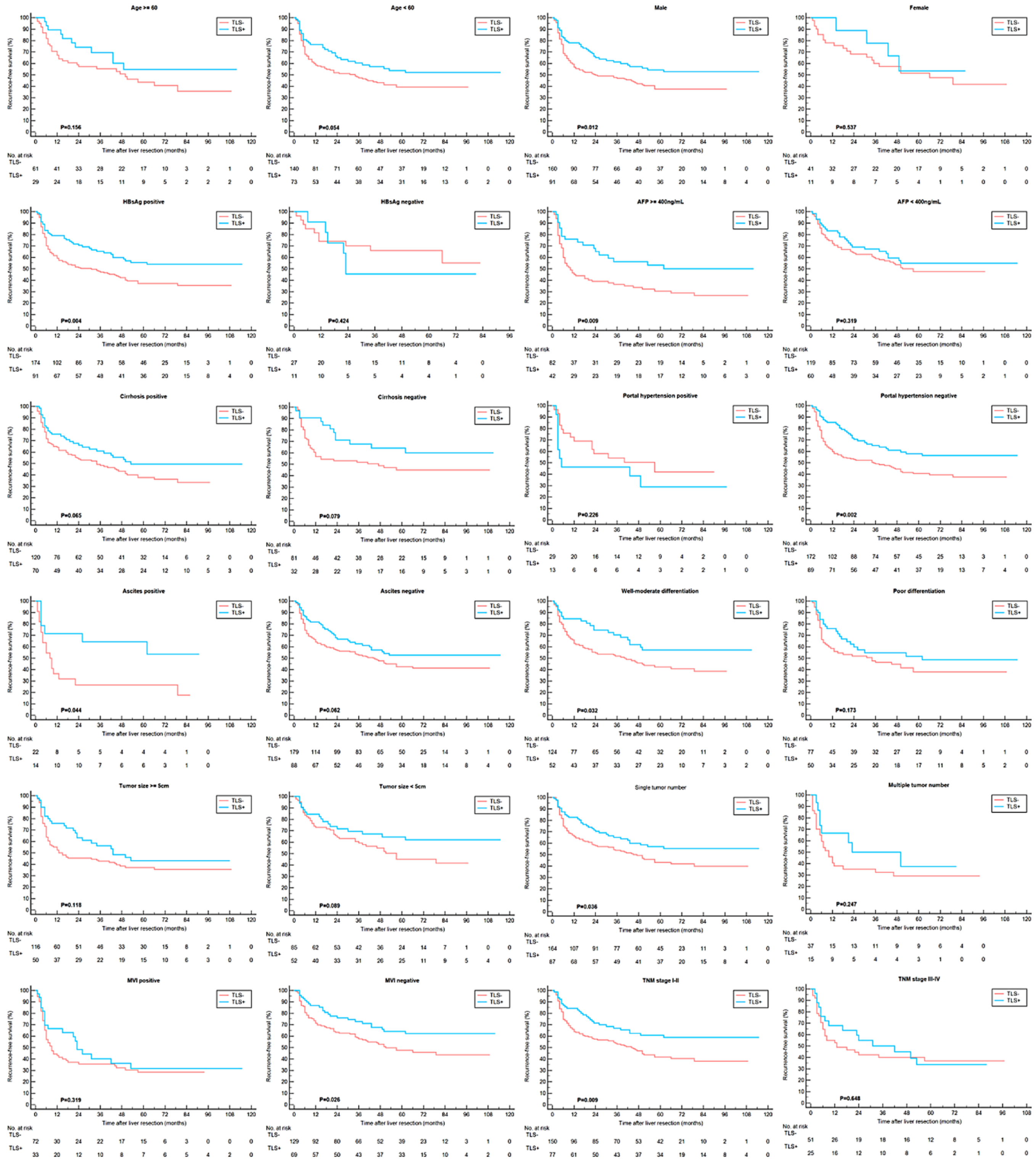

Supplementary Figure 1. Kaplan–Meier curves showing the association between the TLS and RFS stratified by subgroups in the training cohort. TLS, tertiary lymphoid structures; RFS, recurrence-free survival.

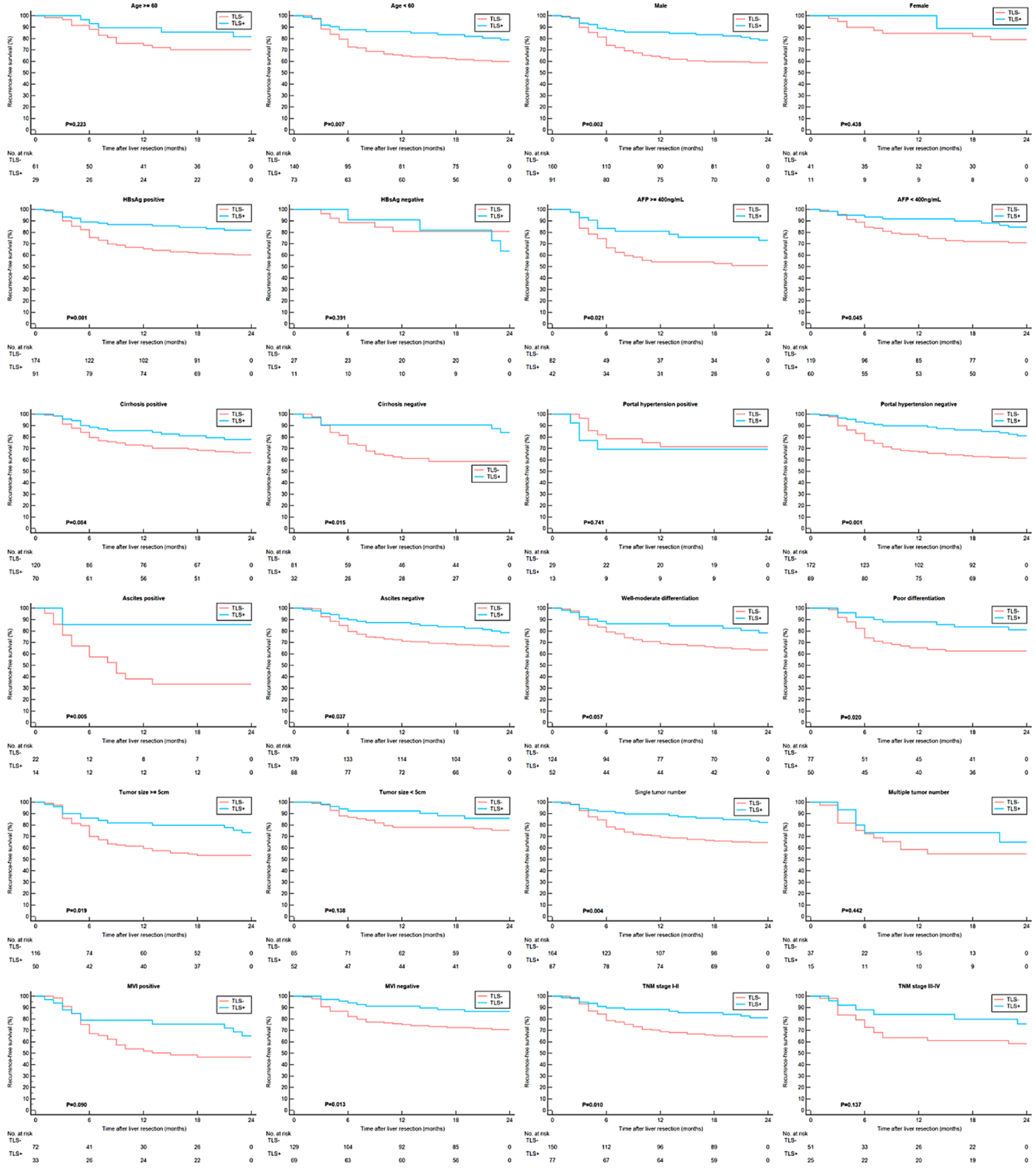

Supplementary Figure 2. Kaplan–Meier curves showing the association between TLS and early RFS stratified by subgroups in the training cohort. TLS, tertiary lymphoid structures; early RFS, early recurrence-free survival.

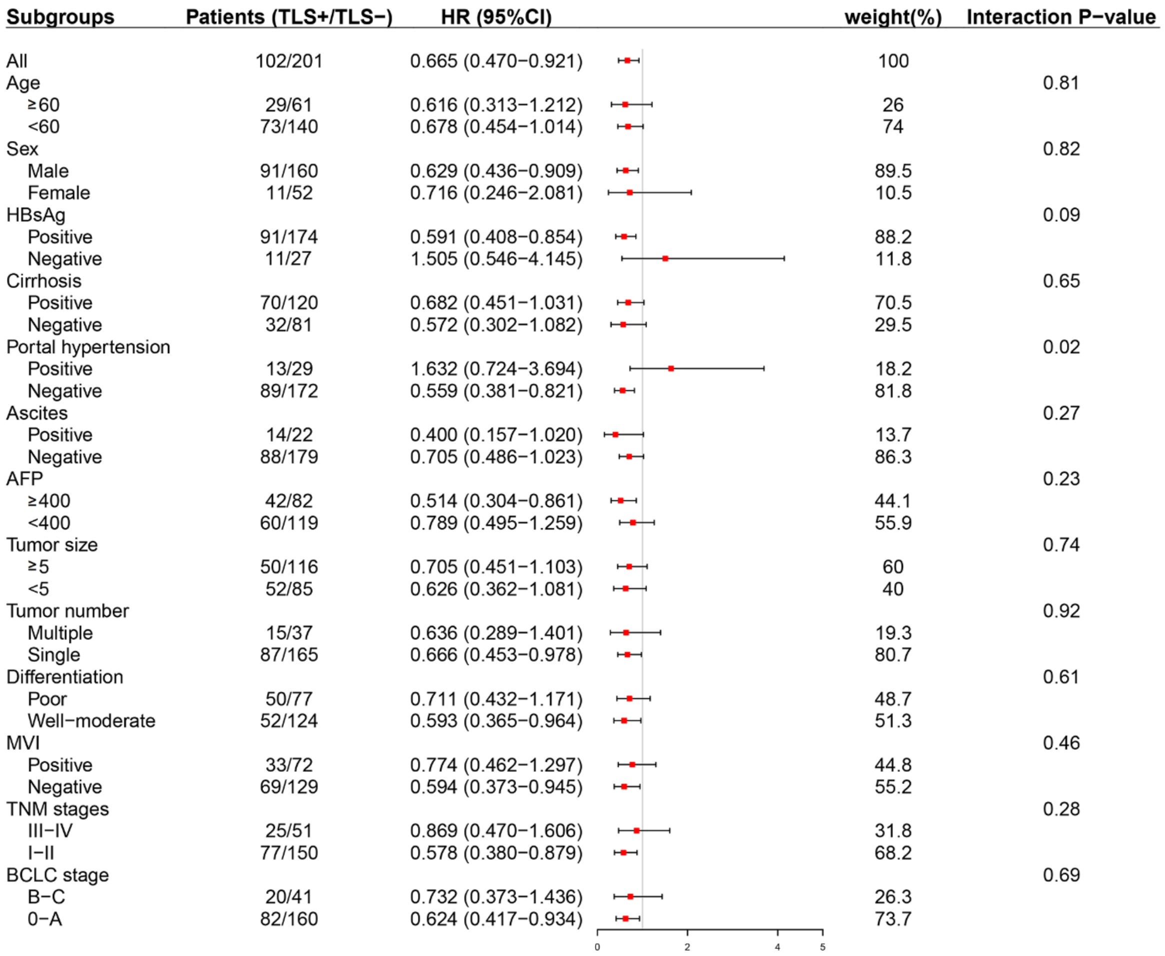

Supplementary Figure 3. Subgroup analysis based on clinicopathological features (recurrence-free survival). TLS, tertiary lymphoid structures; AFP, alpha-fetoprotein; BCLC, Barcelona Clinic of Liver Cancer; TNM, tumor-nodes-metastasis.

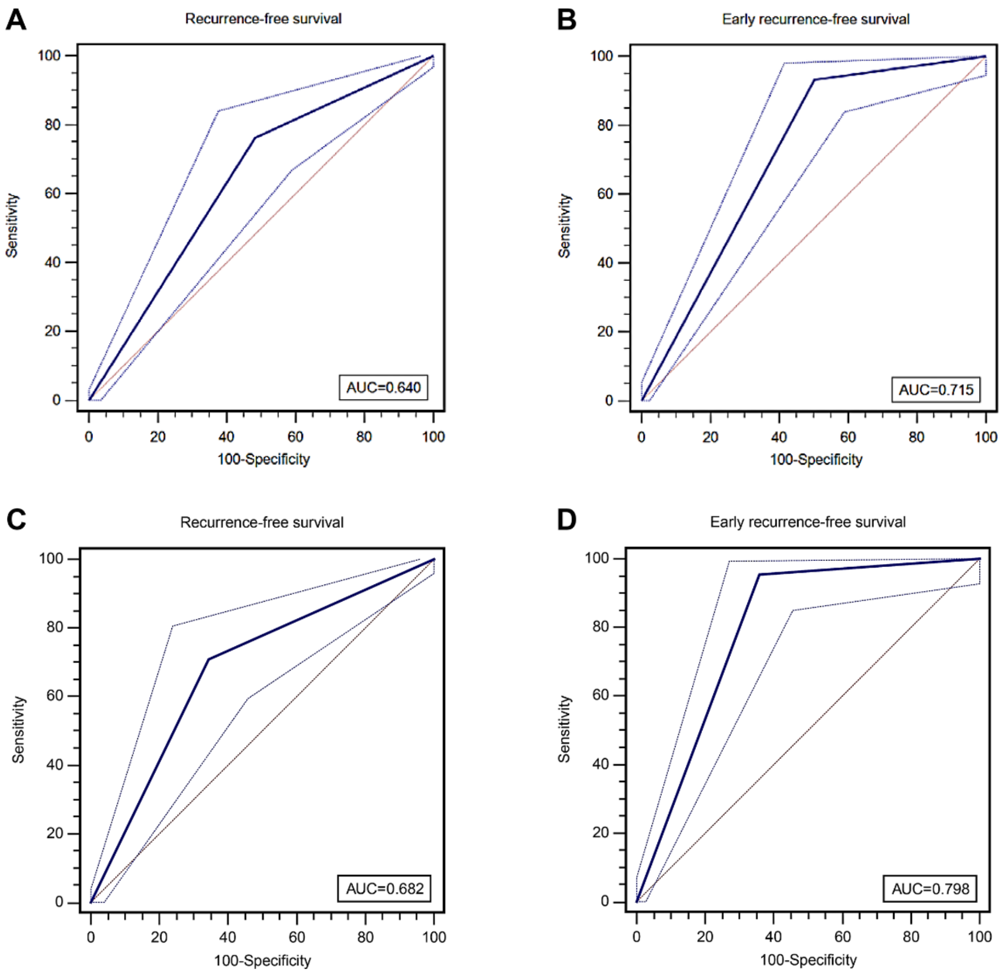

Supplementary Figure 4. ROC curves evaluating the accuracy of TLS in predicting tumor recurrence in training cohort. (**A**) The AUC was 0.64 for TLS in predicting RFS; (**B**) The AUC was 0.715 for TLS in predicting early tumor recurrence; (**C**) The AUC was 0.682 for TLS in predicting RFS for patients with BCLC 0-A stage HCC; (**D**) The AUC was 0.798 for TLS in predicting early tumor relapse for patients with BCLC 0-A stage HCC. ROC, receiver operating characteristic; TLS, tertiary lymphoid structures; AUC, area under the receiver operating characteristic; RFS, recurrence-free survival.
