# Supplementary material for: Existence of intratumoral tertiary lymphoid structures is associated with immune cells infiltration and predicts better prognosis in early-stage hepatocellular carcinoma
Source: Aging (Albany NY). 2020 Feb 22;12(4):3451–72. doi: 10.18632/aging.102821 (PMC7066901; doi:10.18632/aging.102821)
Supplement: Supplementary Tables [file aging-12-102821-s001..pdf]

SUPPLEMENTARY TABLES

Supplementary Table 1. Antibody sources and staining conditions.

Markers	Antibody sources	Cellular location	Species	Antigen retrieval	Dilution
CD3	Abcam, ab16669	Membranous	Rabbit monoclonal	Citrate buffer (pH 6.0)	1:200
CD8	Abcam, ab33786	Membranous	Mouse monoclonal	Citrate buffer (pH 6.0)	1:200
CD20	Abcam, ab9475	Membranous	Mouse monoclonal	Citrate buffer (pH 6.0)	1:200
FOXP3	Abcam, ab20034	Nuclear	Mouse monoclonal	Citrate buffer (pH 6.0)	1:200
CD68	DAKO, M087601-2	Cytoplasmic	Mouse monoclonal	Citrate buffer (pH 6.0)	1:200
PD1	Abcam, ab52587	Membranous	Mouse monoclonal	Citrate buffer (pH 6.0)	1:50
PDL1	CST, #13684	Membranous	Rabbit monoclonal	Tris/EDTA buffer (pH 9.0)	1:200
TIM3	R&D Systems, AF2365-SP	Membranous	Goat polyclonal	Citrate buffer (pH 6.0)	3ug/mL
LAG3	LifeSpan Bioscience, LS-B2237	Membranous	Mouse monoclonal	Citrate buffer (pH 6.0)	1:100

Supplementary Table 2. Correlation between existence of TLS and clinicopathological characteristics of 303 HCC patients in the training cohort.

Variables	All patients (*n*=303)	TLS+ (*n*=102)	TLS- (*n*=201)	*P* Value
Age (year)	51.1±12.5	50.9±13.2	51.3±12.2	0.798
Gender (M/F)	251/52	91/11	160/41	0.038
HBsAg (+/-)	265/38	91/11	174/27	0.585
HBV DNA (0/10^3-10^5/>10^5)	97/105/101	33/39/40	64/66/61	0.717
HCV infection (+/-)	6/297	1/101	5/196	0.442
Cirrhosis (+/-)	190/113	70/32	120/81	0.136
Portal hypertension (+/-)	42/261	13/89	29/172	0.724
Ascites (+/-)	36/267	14/88	22/179	0.576
AFP (≥400/<400) (ng/dL)	124/179	42/60	82/119	1.000
Tumor size (≥5cm/<5cm)	166/137	50/52	116/85	0.184
Tumor number (Multiple/single)	52/251	15/87	37/164	0.421
Differentiation (Poor /Well-Moderate)	127/176	50/52	77/124	0.087
Macrovascular invasion	13 (4.3%)	5 (4.9%)	8 (4.0%)	0.764
Microvascular invasion	105 (34.7%)	33 (32.3%)	72 (35.8%)	0.610
BCLC stages (B-C/0-A)	61/242	20/82	41/160	0.880
TNM stages (III-IV/I-II)	76/227	25/77	51/150	0.889
Early recurrence	90 (29.7%)	20 (19.6%)	70 (34.8%)	0.009
5-year survival	162 (53.5%)	58 (56.9%)	104 (51.7%)	0.464

F: female; M: male; HBV: hepatitis B virus; HCV: hepatitis V virus; AFP: alpha-fetoprotein; BCLC: Barcelona Clinic of Liver Cancer; TNM: tumor-nodes-metastasis; TLS: tertiary lymphoid structures.

Supplementary Table 3. Correlation between existence of TLS and clinicopathological characteristics of 242 BCLC stage 0-A HCC patients in training cohort.

Variables	All patients (*n*=242)	TLS+ (*n*=82)	TLS- (*n*=160)	*P* Value
Age (year)	51.6±12.4	50.1±13.5	52.4±11.8	0.175
Gender (M/F)	197/45	71/11	126/34	0.165
HBsAg (+/-)	212/30	75/7	137/23	0.223
HBV DNA (0/10^3-10^5/>10^5)	82/87/73	30/36/16	52/51/57	0.076
HCV infection (+/-)	5/237	1/81	4/156	0.667
Cirrhosis (+/-)	150/92	54/28	96/64	0.408
Portal hypertension (+/-)	34/208	8/74	26/134	0.181
Ascites (+/-)	29/213	13/69	16/144	0.212
AFP (≥400/<400) (ng/dL)	94/148	33/49	61/99	0.788
Tumor size (≥5cm/<5cm)	123/119	36/46	87/73	0.137
Differentiation (Poor /Well-Moderate)	102/140	40/42	62/98	0.136
Microvascular invasion	67 (27.7%)	22 (26.8%)	45 (28.1%)	0.832
Early recurrence	67 (27.7%)	13 (15.9%)	54 (33.8%)	0.004
5-year survival	140 (57.9%)	50 (61.0%)	90 (56.3%)	0.499

F: female; M: male; HBV: hepatitis B virus; HCV: hepatitis C virus; AFP: alpha-fetoprotein; BCLC: Barcelona Clinic of Liver Cancer; TLS: tertiary lymphoid structures.

Supplementary Table 4. Comparison of clinicopathological characteristics between patients with BCLC 0-A stage HCC in training cohort and patients in validation cohort.

Variables	Training cohort (*n*=242)	Validation cohort (n=159)	*P* Value
Age (year)	51.6±12.4	51.2±12.6	0.975
Gender (M/F)	197/45	132/27	0.790
HBsAg (+/-)	212/30	140/19	0.894
HBV DNA (0/10^3-10^5/>10^5)	82/87/73	50/57/52	0.544
HCV infection (+/-)	5/237	3/156	1.000
Cirrhosis (+/-)	150/92	98/61	0.944
Portal hypertension (+/-)	34/208	22/137	0.952
Ascites (+/-)	29/213	20/139	0.859
AFP (≥400/<400) (ng/dL)	94/148	64/95	0.778
Tumor size (≥5cm/<5cm)	123/119	84/75	0.695
Differentiation (Poor /Well-Moderate)	102/140	68/91	0.903
Microvascular invasion	67 (27.7%)	50 (31.4%)	0.419
Early recurrence	67 (27.7%)	45 (28.3%)	0.084
5-year survival	140 (57.9%)	80 (50.3%)	0.139

F: female; M: male; HBV: hepatitis B virus; HCV: hepatitis C virus; AFP: alpha-fetoprotein; BCLC: Barcelona Clinic of Liver Cancer; TNM: tumor-nodes-metastasis; TLS: tertiary lymphoid structures; N/A: not available.

Supplementary Table 5. Correlation between existence of TLS and clinicopathological characteristics in the validation cohort.

Variables	All patients (*n*=159)	TLS+ (*n*=54)	TLS- (*n*=105)	*P* Value
Age (year)	51.2±12.6	49.2±13.5	52.2±12.1	0.157
Gender (M/F)	132/27	46/8	86/19	0.662
HBsAg (+/-)	140/19	75/7	137/23	0.223
HBV DNA (0/10^3-10^5/>10^5)	50/57/52	16/27/11	34/30/41	0.015
HCV infection (+/-)	3/156	1/53	2/103	0.981
Cirrhosis (+/-)	98/61	30/24	68/37	0.488
Portal hypertension (+/-)	22/137	7/47	15/90	0.819
Ascites (+/-)	20/139	8/46	12/93	0.616
AFP (≥400/<400) (ng/dL)	64/95	23/31	41/64	0.734
Tumor size (≥5cm/<5cm)	84/75	22/32	62/43	0.137
Differentiation (Poor /Well-Moderate)	68/91	28/26	40/65	0.127
Microvascular invasion	50 (31.4%)	17 (31.4%)	33 (31.4%)	1.000
Early recurrence	45 (28.3%)	10 (18.5%)	35 (33.3%)	0.014
5-year survival	80 (50.3%)	29 (53.7%)	51 (48.6%)	0.616

F: female; M: male; HBV: hepatitis B virus; HCV: hepatitis C virus; AFP: alpha-fetoprotein; BCLC: Barcelona Clinic of Liver Cancer; TLS: tertiary lymphoid structures

Supplementary Table 6. Analysis for overall survival and recurrence-free survival using the univariate and multivariate Cox proportional hazards regression model in the training cohort.

Variables	Overall survival		Recurrence-free survival	
	HR (95%CI)	P value	HR (95%CI)	P value
Age	0.997(0.985-1.009)	0.606	0.997(0.985-1.009)	0.637
Gender (F/M)	0.853(0.569-1.278)	0.440	0.788(0.514-1.208)	0.275
HBsAg (+/-)	1.529(0.927-2.522)	0.096	1.484(0,885-2.488)	0.134
HBV DNA (>10^5/10^3-10^5/0)	1.615(1.334-1.957)	<0.001	1.301(1.072-1.579)	0.008
HCV infection (+/-)	3.451(1.524-7,813)	<0.001	1.976(0.732-5.336)	0.179
Cirrhosis (+/-)	1.142(0.839-1.555)	0.399	1.203(0.868-1.667)	0.268
Portal hypertension (+/-)	1.131(0.723-1.771)	0.589	1.237(0.807-1.896)	0.328
Ascites (+/-)	1.304(0.848-2.007)	0.227	1.708(1.092-2.672)	0.019
AFP (≥400/<400)	1.193(0.884-1.610)	0.248	1.661(1.217-2.268)	0.001
Tumor size (≥5/<5)	1.799(1.322-2.450)	<0.001	1.589(1.156-2.185)	0.004
Tumor number (Multiple/single)	1.750(1.225-2.500)	0.002	1.737(1.189-2.539)	0.004
Differentiation (Poor /Well-moderate)	1.175(0.872-1.583)	0.289	1.129(0.825-1.544)	0.448
Macrovascular invasion	3.549(1.909-6.600)	<0.001	3.272(1.761-6.079)	<0.001
Microvascular invasion	2.168(1.608-2.923)	<0.001	2.083(1.521-2.851)	<0.001
BCLC (B-C/0-A)	1.930(1.377-2.706)	<0.001	1.811(1.265-2.593)	0.001
TNM (III-IV/I- II)	1.333(0.961-1.850)	0.086	1.455(1.033-2.050)	0.032
TLS (+/-)	0.754(0.545-1.043)	0.088	0.665(0.470-0.921)	0.014

F: female; M: male; HBV: hepatitis B virus; HCV: hepatitis V virus; AFP: alpha-fetoprotein; BCLC: Barcelona Clinic of Liver Cancer; TNM: tumor-nodes-metastasis; TLS: tertiary lymphoid structures; HR, hazard ratio; CI, confidence interval.

Supplementary Table 7. Analysis for overall survival and recurrence-free survival using the univariate and multivariate Cox proportional hazards regression model for BCLC stage 0-A HCC in training cohort.

Variables	Overall survival				Recurrence-free survival			
	Univariate analysis		Multivariate analysis		Univariate analysis		Multivariate analysis	
	HR (95%CI)	P value	HR (95%CI)	P value	HR (95%CI)	P value	HR (95%CI)	P value
Age	0.999(0.986-1.013)	0.906			1.002(0.989-1.014)	0.795		
Gender (F/M)	0.808(0.511-1.278)	0.361			0.806(0.539-1.204)	0.292		
HBsAg (+/-)	1.570(0.867-2.844)	0.137			0.974(0.615-1.543)	0.912		
HBV DNA ($>10^5/10^3$-$10^5/0$)	1.578(1.263-1.972)	<0.001	1.497(1.194-1.878)	<0.001	1.240(1.017-1.511)	0.033	1.163(0.952-1.419)	0.139
HCV infection (+/-)	3.330(1.357-8.174)	0.009	3.187(1.278-7.949)	0.013	1.899(0.703-5.133)	0.206		
Cirrhosis (+/-)	1.165(0.814-1.667)	0.405			0.880(0.643-1.203)	0.421		
Portal hypertension (+/-)	0.920(0.551-1.534)	0.748			0.929(0.587-1.469)	0.752		
Ascites (+/-)	1.410(0.866-2.295)	0.167			1.866(1.194-2.915)	0.006	1.632(1.049-2.538)	0.030
AFP (≥400/<400)	1.101(0.663-1.350)	0.760			0.984(0.715-1.354)	0.920		
Tumor size (≥5/<5)	2.041(1.429-2.916)	<0.001	1.740(1.208-2.507)	0.003	1.607(1.178-2.194)	0.003	1.541(1.194-2.114)	0.007
Differentiation (Poor /Well-Moderate)	1.278(0.905-1.804)	0.164			1.114(0.815-1.523)	0.497		
Microvascular invasion	1.892(1.319-2.714)	0.001	1.752(1.209-2.539)	0.003	1.272(0.899-1.799)	0.174		
TLS (+/-)	0.803(0.553-1.167)	0.251			0.651(0.463-0.914)	0.013	0.655(0.463-0.926)	0.017

F: female; M: male; HBV: hepatitis B virus; HCV: hepatitis C virus; AFP: alpha-fetoprotein; BCLC: Barcelona Clinic of Liver Cancer; TLS: tertiary lymphoid structures; HR, hazard ratio; CI, confidence interval.

Supplementary Table 8. Analysis for early and late recurrence-free survival using the univariate and multivariate Cox proportional hazards regression model for BCLC stage 0-A HCC in the training cohort.

Variables	Early recurrence-free survival				Late recurrence-free survival			
	Univariate analysis		Multivariate analysis		Univariate analysis		Multivariate analysis	
	HR (95%CI)	P value	HR (95%CI)	P value	HR (95%CI)	P value	HR (95%CI)	P value
Age	0.988(0.970-1.007)	0.227			1.008(0.989-1.026)	0.408		
Gender (F/M)	0.544(0.260-1.138)	0.106			1.134(0.676-1.903)	0.633		
HBsAg (+/-)	1.321(0.604-2.890)	0.486			0.805(0.424-1.529)	0.508		
HBV DNA ($>10^5/10^3-10^5/0$)	1.362(1.008-1.841)	0.044	1.225(0.903-1.662)	0.192	1.259(0.937-1.693)	0.127		
HCV infection (+/-)	1.825(0.447-7.459)	0.402			1.324(0.184-9.552)	0.781		
Cirrhosis (+/-)	0.730(0.451-1.181)	0.200			0.921(0.587-1.447)	0.722		
Portal hypertension (+/-)	0.801(0.383-1.676)	0.556			0.818(0.408-1.638)	0.570		
Ascites (+/-)	2.512(1.360-4.640)	0.003	2.053(1.120-3.762)	0.020	1.046(0.481-2.274)	0.911		
AFP (≥400/<400)	1.625(1.006-2.625)	0.047	1.393(0.851-2.279)	0.187	0.617(0.380-1.003)	0.052		
Tumor size (≥5/<5)	2.315(1.389-3.857)	0.001	1.888(1.105-3.224)	0.020	1.191(0.763-1.860)	0.441		
Differentiation (Poor /Well-Moderate)	1.084(0.668-1.757)	0.745			0.900(0.567-1.430)	0.656		
Microvascular invasion	2.051(1.258-3.343)	0.004	1.733(1.051-2.857)	0.031	1.574(0.867-2.858)	0.136		
TLS (+/-)	0.415(0.226-0.760)	0.004	0.410(0.220-0.761)	0.005	0.711(0.443-1.141)	0.157		

F: female; M: male; HBV: hepatitis B virus; HCV: hepatitis V virus; AFP: alpha-fetoprotein; BCLC: Barcelona Clinic of Liver Cancer; TLS: tertiary lymphoid structures; HR, hazard ratio; CI, confidence interval.
